# Mental Health Problems Among Adolescents in Sweden from 1995 to 2011: The Role of Immigrant Status and the Proportions of Immigrant Adolescents in Their Surrounding Community

**DOI:** 10.1007/s10903-019-00951-0

**Published:** 2019-12-11

**Authors:** Yunhwan Kim, Brittany E. Evans, Curt Hagquist

**Affiliations:** Centre for Research on Child and Adolescent Mental Health, Karlstad University, 1D 349B, Universitetsgatan 2, Karlstad, SE 651 88 Sweden

**Keywords:** Immigrant status, Adolescents, Mental health, Trend, Proportion of immigrants

## Abstract

We compared the mental health of native and immigrant adolescents in Sweden from 1995 to 2011 and examined whether the municipality-level proportion of immigrant adolescents moderated the association between individual-level immigrant status and mental health. The sample (14,189 adolescents aged 15–16) was obtained from a repeated cross-sectional study conducted from 1995 to 2011. Adolescent self-report data (gender, immigrant status, economic situation, and mental health) and municipality-level data (proportion of immigrant adolescents) were used in multilevel linear regression analyses. Immigrant adolescents reported more mental health problems than their native Swedish peers. The long-term trend in mental health problems did not differ between the two groups. The association between individual immigrant status and mental health did not differ according to the municipality-level rate of immigrant adolescents. These findings highlight the need for public health attention to and efforts to address immigrant adolescents’ mental health problems in Sweden.

## Background

Immigration is increasingly a part of reality in many societies today and the implications of immigrant populations’ well-being for themselves and the host societies are correspondingly becoming more important [1–3]. Comparing mental health between immigrant adolescents and their native counterparts enables us to gauge how well immigrants are doing; an area where research is still sparse [4]. In this study, we compared the mental health of immigrant adolescents in Sweden and their native peers. We also examined whether the association between immigrant status and mental health was moderated by the proportion of immigrant adolescents in their surrounding communities.

### Mental Health of Immigrant Adolescents

Research on mental health problems among immigrants is inconclusive [5]. Immigrant adolescents have additional challenges to handle than their native peers at the individual level (e.g., events and transitions experienced before, during, and after the immigration processes) and structural level (e.g., unfavorable opportunities in the host society, lack of access to social benefits, discrimination). Hence, immigrant adolescents may tend to report worse mental health than their native peers. Indeed, this was the case in, for example, Norway, Spain and Italy [4, 6, 7]. A recent study conducted in ten European countries also reported more mental health problems among immigrant adolescents than their native peers [8].

It has also been observed that many immigrants generally do well [1, 9]. Furthermore, some scholars have discussed an “immigrant paradox”, where immigrant adolescents present more favorable mental health than their native peers [10, 11]. Similarly, the resilience perspective highlights the potential of successful immigrant adolescents [12]. Aspirations for success, family ties, reasons for immigration, and integration policies in the host society may be critical factors regarding mental health among immigrant adolescents [1, 10, 13–15]. Swedish research on this issue has also yielded mixed findings. While some reported worse mental health among immigrant adolescents than native peers [16, 17], others reported better mental health among immigrant adolescents [18, 19].

### Time Trends in Mental Health

Time-trend studies indicated that mental health problems have increased among Swedish adolescents in the past few decades [20, 21]. Changing societal structures often influence different groups in different ways [22], hence the trends in mental health between immigrant adolescents and their native peers may not be parallel. During the past few decades, the size and living conditions of immigrant populations have changed substantially in many countries, including Sweden [23, 24]. No study to date has compared the long-term time trends in immigrant and native adolescents’ mental health problems.

### Immigrant Population in the Surrounding Community

Changes in the proportion of immigrants in the surrounding community translate to different living conditions for individual immigrants. Thus, the proportion of immigrants in the community may moderate the association between individual immigrant status and mental health problems. A higher proportion of immigrants in a community may contribute favorably to individual immigrants’ adjustment. Indeed, from a random household sample in New Zealand, it was found that having more intercultural contacts (defined by more frequent interactions with immigrants and lower levels of intergroup anxiety) was linked to more favorable attitudes towards immigrants both directly and indirectly through decreased perceived threat related to immigrants [25]. Also, from a longitudinal sample of Greek secondary school students, it was found that when the proportion of immigrants in their classroom was higher compared to lower, immigrant adolescents were more popular; however, their mental health was not better [26].

### Economic Status as a Confounding Factor

Individuals’ economic situation is often considered a confounding factor regarding mental health among immigrant populations. Immigrant adolescents are often economically disadvantaged, and Sweden was not an exception to this [17]. Furthermore, economic strain may worsen mental health, implying that economic reasons might underlie mental health problems among immigrant adolescents. This was empirically supported in Italy and the Netherlands [4, 27]. In Sweden, Carlerby et al. [17], using a nationally representative adolescent sample, evaluated whether socioeconomic status contributed to the mental health gap between immigrant and native adolescents. The results indicated that the mental health gap between native Swedish girls and girls with a foreign background was unaffected by the girls’ socioeconomic status. However, socioeconomic status was one of five socio-demographic control variables that were simultaneously entered into the regression model, therefore it was not possible to evaluate the independent effect of socioeconomic status on the mental health gap.

### The Current Study

The current study aimed to answer the following research questions after controlling for the effects of adolescents’ economic situation [5] and gender [20].

1. Do immigrant adolescents in Sweden report worse mental health than their native peers?
2. Does the time trend in mental health from 1995 to 2011 differ between immigrant adolescents and their native peers?
3. Do immigrant adolescents report better mental health when living in a community with higher rates of immigrant adolescents than one with lower rates?

## Method

### Participants

The study sample was obtained from a repeated cross-sectional study conducted every 3 to 4 years between 1988 and 2011 (8-times), the Young in Värmland (YiV) study. The YiV study targeted all students in grade 9 (aged 15–16) in a region of Sweden (Värmland; 16 municipalities). This study used a subsample due to data availability. First, data on immigrant status was not available for the first two investigation years (1988 and 1991). We also excluded data from two municipalities that did not participate in the study in 1995. This reduced the sample size from 23,167 to 14,809. About 4% of the remaining sample (*n *= 620) was excluded due to missing data, therefore the size of the final sample was 14,189.

### Procedure

Data were collected during regular school hours during the spring semester of the academic year in grade 9. Before the data collection, the parents of all eligible participants were informed of the study. Student participation was thus voluntary. They completed a self-report questionnaire in the classroom and submitted it to school personnel in a sealed envelope. The data collection procedure followed the research ethics principles in humanistic-social science research stipulated by the Swedish Research Council. The questionnaire and the principles guiding the data collections in 2005 and onwards were reviewed by the Ethics Committee at Karlstad University, Sweden.

### Measures

#### Mental Health

We measured adolescents’ mental health with the psychosomatic problems (PSP) scale which showed a good validity and reliability in a previous psychometric study that used the same data as the current study [28]. Adolescents reported how often they had experienced each of the 8 psychosomatic symptoms during the current school year on a 5-point scale (*never*, *seldom*, *sometimes*, *often*, and *always*): difficulty concentrating, problems sleeping, headaches, stomachaches, feeling tense, poor appetite, feeling sad, and feeling giddy. Psychometric analysis based on the Rasch model assigned each adolescent a location value on a logit scale where a higher value indicates a greater degree of psychosomatic problems. We considered psychosomatic problems as a relevant indicator of mental health problems as this indicator has previously been used to investigate long-term trends in mental health among Swedish adolescents as well as group differences in this trend e.g., [20, 29, 30].

#### Immigrant Status and Proportion of Immigrants In The Community

**Fig. 1:**
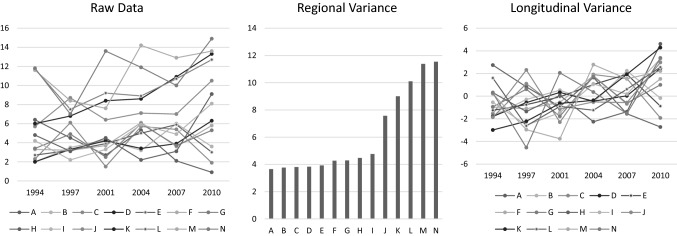
The proportion of students with an immigration background by municipality and year. The variation in raw data was decomposed into regional and longitudinal variance. The regional variance was first obtained from raw data by averaging the values of the six assessment points for each municipality. Then, the longitudinal variance was obtained by substracting the regional variance from the raw data

Adolescents reported the birth place(s) of their parents and were considered to have an immigration background if one or both parents were born outside Sweden. The proportion of first and second generation immigrants in each municipality during each year of investigation was calculated using registry data of all grade 9 students in Värmland. Considering that the individual-level data was collected during the spring semester and in order to ensure that events that occurred after that did not bias our results, we used municipality-level data from a year previous to each data collection year. As this data varies by both municipality and year, following Fairbrother’s [31] method, we decomposed it into both regional and longitudinal variance (Fig. 1).

#### Control Variables

We controlled for adolescents’ gender and their unaffordability of daily leisure activities as a proxy of their economic situation. Regarding the latter, the adolescents answered four questions asking how often in the past month, due to financial constraints, they were not able to go to: a movie, a concert and listen to live music, a sports event, and a disco or dance club. The response options were *never*, *occasionally*, and *several times*. The indicator of economic situation was created by summing and then standardizing the scores of these four items. The unaffordability of daily leisure activities has explained mental health among Swedish adolescents in the context of cross-sectional [32] as well as time trend studies [30].

### Analysis

After conducting a descriptive analysis of the study variables, the main analyses consisted of multilevel linear regression with individuals (level 1) nested in years of investigation (level 2) nested in municipality (level 3). We estimated a series of models and compared each model to the previous using a likelihood ratio test (LRT). First, to examine the difference in the degree of mental health problems between immigrant and native adolescents (RQ1), we entered individual-level immigrant status into the analysis as the predictor of mental health problems (Model 2) and compared this model to the null model (Model 1). Then, in order to examine whether the Model 2 results remained after accounting for potential confounders, we added adolescents’ gender and unaffordability and investigation years to the model (Model 3). Next, in order to examine whether the trend in mental health problems differed between immigrant and native adolescents (RQ2), we tested the interaction between individual-level immigrant status and investigation years (Model 4). Finally, in order to examine whether the proportion of immigrants in the community modified the association between individual-level immigrant status and mental health (RQ3), municipality-level proportion of immigrant students and its interaction with individual level-immigrant status were added to the model (Model 5).

## Results

### Descriptive Results

**Fig. 2:**
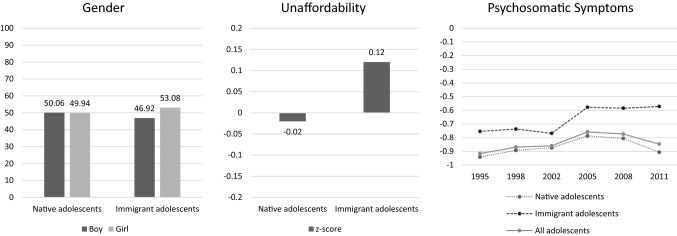
Descriptive statistics. A significant difference was found between native and immigrant adolescents in terms of gender composition (χ^2^(1) = 6.78, *p* < 0.01) and unaffordability (*t*(13722) = − 5.94, *p* < 0.001). The scores of the psychosomatic symptoms are the logit values obtained from the Rasch psychometric analysis based on 8 psychosomatic symptoms (see the method section); a higher value indicates a greater degree of psychosomatic symptoms

Descriptive statistics of the study variables are shown in Figs. 1 and 2. While there are noticeable differences in the proportions of adolescent immigrants between municipalities, most municipalities experienced a similar increase in this proportion over the investigation period (Fig. 1). Across municipalities, the average proportions of immigrant students were 5.6, 5.2, 5.4, 6.7, 6.4, and 7.8% in 1994, 1997, 2001, 2004, 2007, and 2010, respectively. Figure 2 illustrates that immigrant and native adolescents differed significantly in terms of gender, χ^2^(1) = 6.78, *p* < 0.01, and unaffordability, *t*(13722) = − 5.94, *p* < 0.001.

The prevalence of immigrant adolescents’ mental health problems was higher than native adolescents across all investigation years (Fig. 2). Overall, the prevalence of mental health problems increased until 2005 and decreased slightly thereafter. However, when the trend was differentiated according to adolescents’ immigration background, the pattern after 2005 among immigrant adolescents diverged: their level of mental health problems continued to increase, although by very little.

### Mental Health Problems Among Immigrant and Native Adolescents

Adding individual-level immigrant status to the null model significantly improved the model fit (Table 1). The results from Model 2 showed that immigrant adolescents reported significantly higher levels of mental health problems than native adolescents (*r* = 0.21, *p* < 0.001). In Model 3 we included adolescents’ gender and unaffordability and investigation years, which significantly improved model fit compared to Model 2. Specifically, the level of mental health problems were significantly higher after 2002 compared to the reference year 1995. Also, the level of mental health problems was higher among girls and among those who experienced unaffordability of leisure activities more often. Most importantly, after controlling for these factors, the difference between immigrant and native adolescents remained significant (*r* = 0.15, *p* < 0.001). The regression coefficients of immigrant status when each control variable was entered separately were 0.19, 0.21, and 0.16 (all *p*s < 0.001), respectively for gender, investigation years, and economic situation.

**Table 1:** Multilevel linear regression models predicting psychosomatic symptoms

	Model 1	Model 2	Model 3	Model 4	Model 5
DV = Psychosomatic symptoms (*M* = − 0.83, *SD* = 1.21)					
Fixed effects					
Constant	− 0.83 [− 0.87, − 0.78]***	− 0.86 [− 0.90, − 0.81]***	− 1.27 [− 1.33, − 1.20]***	− 1.27 [− 1.34, − 1.19]***	− 1.23 [− 1.33, − 1.12]***
Immigrant status (0 = native/1 = immigrants)		0.21 [0.15, 0.27]***	0.15 [0.10, 0.20]***	0.14 [− 0.00, 0.29]^*p* = 0.06^	0.11 [− 0.06, 0.27]
Year (c.f. 1995)					
1998			0.04 [− 0.04, 0.12]	0.05 [− 0.04, 0.14]	0.04 [− 0.03, 0.12]
2002			0.13 [0.05, 0.21]***	0.14 [0.05, 0.23]**	0.13 [0.05, 0.21]***
2005			0.24 [0.16, 0.32]***	0.24 [0.15, 0.33]***	0.25 [0.17, 0.33]***
2008			0.28 [0.20, 0.35]***	0.27 [0.18, 0.36]***	0.29 [0.21, 0.37]***
2011			0.18 [0.10, 0.26]***	0.17 [0.08, 0.27]***	0.21 [0.12, 0.31]***
Gender (0 = boy/1 = girl)			0.54 [0.51, 0.58]***	0.54 [0.51, 0.58]***	0.54 [0.51, 0.58]***
Unaffordability			0.26 [0.24, 0.28]***	0.26 [0.24, 0.28]***	0.26 [0.24, 0.28]***
Year*immigrant status					
1998				− 0.02 [− 0.22, 0.19]	
2002				− 0.10 [− 0.31, 0.11]	
2005				0.02 [− 0.19, 0.22]	
2008				0.01 [− 0.20, 0.21]	
2011				0.06 [− 0.15, 0.26]	
Proportion (regional)					− 0.01 [− 0.02, 0.01]
Proportion (regional) by Immigrant status					0.01 [− 0.02, 0.03]
Proportion (longitudinal)					− 0.01 [− 0.03, 0.00]
Proportion (longitudinal) by Immigrant status					0.02 [− 0.01, 0.05]
Random effects					
Municipality: Intercept	0.00 [0.00, 0.02]	0.00 [0.00, 0.02]	0.00 [0.00, 0.01]	0.00 [0.00, 0.01]	0.00 [0.00, 0.01]
Municipality: slope					0.00 [0.00, 0.83]
Year: intercept	0.01 [0.00, 0.02]	0.01 [0.00, 0.02]	0.00 [0.00, 0.02]	0.00 [0.00, 0.00]	0.00 [0.00, 0.00]
Year: slope				0.00 [0.00, 0.02]	0.00 [0.00, 0.02]
Residual	1.46 [1.43, 1.50]	1.46 [1.42, 1.49]	1.31 [1.28, 1.34]	1.31 [1.28, 1.34]	1.31 [1.28, 1.34]
Model fit indices					
AIC	44244.84	44197.01	42697.19	42704.32	42702.48
BIC	44274.95	44234.64	42787.51	42839.81	42837.96
LRT comparison		To model 1 χ^2^(1) = 49.83***	To model 2 χ^2^(7) = 1513.82***	To model 3 χ^2^(6) = 4.87	To model 3 χ^2^(6) = 6.71

ICCs for the null model were 0.003 [0.001, 0.012] and 0.008 [0.004, 0.016], respectively for municipality and year level. Given the descriptive diverging trend between immigrant and native adolescents from 2005 shown in Fig. 2, we conducted some sets of parallel analyses. First, we conducted the same analysis but without control variables. The results did not reveal any difference in the mental health trend between immigrant and native adolescents both when the analysis was done for all adolescents together and when the analysis was done separately for each gender. Second, we added a three-way interaction term between investigation years and gender to the Model 4 above. Again, the mental health trend did not differ between immigrant and native adolescents

**p < 0.01, ***p < 0.001

### Time Trend in Mental Health Among Immigrant and Native Adolescents

The difference in the time trend in mental health problems between immigrant and native adolescents was examined by testing the interaction of individual-level immigrant status and investigation years. The addition of the interaction term (Model 4) did not produce a better model fit than Model 3. In addition, none of the interaction terms were significant, indicating that the trend in mental health problems over time did not differ between immigrant and native adolescents.

### Proportion of Immigrants in the Surrounding Community

When we added the municipality-level proportion of immigrants (regional and longitudinal components) and the interactions with individual-level immigrant status (Model 5), the model fit was not significantly improved compared to Model 3. For both the regional and longitudinal variation in the proportion of immigrant students, the interaction effects with individual-level immigrant status were not significant. This indicates that the association between individual-level immigrant status and mental health problems did not differ according to the municipality-level proportion of immigrant students.

## Discussion

Our findings indicate that individual-level immigrant status was a risk factor for mental health problems among immigrant adolescents in Sweden from 1995 to 2011. However, the time trends in mental health problems did not differ between immigrant and native adolescents. Furthermore, the gap in mental health problems between immigrant and native adolescents did not differ according to the municipality-level proportion of immigrant students.

### Mental Health Among Immigrant and Native Adolescents

Immigrant adolescents presented a higher degree of mental health problems than native adolescents. Thus, between the two competing hypotheses on immigrant adolescents’ mental health in comparison to their native peers of worse versus the same or better, our finding supports the former. This indicates a need for public health attention to the mental health of immigrant adolescents in Sweden who, despite their poorer mental health than natives, do not necessarily seek and receive more treatment [16].

Consistent with Stevens and Vollebergh [5] ’s evaluation, the difference in mental health problems between immigrant and native adolescents remained significant after accounting for the effects of economic situation, a potential confounder for worse mental health among immigrant populations. This indicates that economic reasons may not underlie the mental health gap between immigrant and native adolescents in Sweden. Once immigrants are settled in the host society, other factors besides economic factors may contribute to their adjustment [33]. Two non-economic considerations are relevant to the Swedish context that may contribute to the mental health gap between immigrant and native adolescents.

The first concerns migration-related factors that may be disadvantageous to immigrant adolescents’ mental health in the long run. Kirmayer et al. [34] exemplified such factors that include exposures to traumatic experiences and harsh living conditions and separation from significant others. These indeed are relevant to the current study as most immigrants in Sweden consist of refugees, asylum seekers, and/or unskilled labor workers [5]. We suggest that other European studies indicating poorer mental health among immigrant adolescents compared to native peers e.g., [4, 6–8] may share this underlying reason to some degree.

The second consideration concerns the social climate that reflects, and is reflected in, attitudes and government policies toward immigrants in the host society. Favorable attitudes towards immigrants and integration-orientated policies in the host society usually have positive implications for mental health among immigrants [33, 35, 36]. Thus, our observation of poorer mental health among immigrant adolescents compared to natives was unexpected given that the Swedish society has maintained an accepting atmosphere and favorable attitudes towards immigrants [37, 38]. However, it has also been suggested that Swedish society is majority-oriented, which may structurally burden immigrant adolescents and affect their mental health [17].

### Time Trends in Mental Health Problems Between Immigrant and Native Adolescents

Given potentially distinctive impacts of societal changes on the mental health of different groups, previous studies explored the time trends in adolescent mental health according to gender, age, socio-economic status, and family structure [20, 22, 29, 39]. To our knowledge, this is the first study that empirically tested whether the time trends in mental health problems differed according to immigrant status in Sweden. Our evidence indicates that the increased trend of mental health problems among Swedish adolescents held for both immigrant and native adolescents.

Although statistically not significant, the descriptive pattern (Fig. 2) suggests that, whereas mental health problems decreased from 2005 onwards among native adolescents, mental health problems continued to increase among immigrant adolescents. Immigration has become an increasingly important issue after our last investigation year (i.e., 2011) and, since 2015, has been ranked as the social issue of the utmost importance among EU member states [40]. Clearly, the trends in mental health after our investigation period require further investigation.

### Proportion of Immigrants in the Surrounding Community

In this study, the association between immigrant status and adolescents’ mental health did not differ according to the proportion of immigrant adolescents in their community. That is, immigrant adolescents’ mental health was not more favorable when living in municipalities in which more adolescents had an immigration background. This is consistent with a previous study that reported no significant interaction between individual-level immigrant status and the proportion of immigrant students in the classroom in predicting mental health [26].

Perhaps, the positive influences of increasing immigrant populations in the community extend only to some areas of adolescents’ lives that are related to mental health problems such as peer popularity [26] but not to mental health problems directly. Similarly, a recent Swedish study using registered population data reported that immigrant adolescents’ educational outcomes were not much benefited from higher proportions of immigrant classmates [41]. Another possibility is that any positive influences may have been offset by other negative, counteracting influences. As the Swedish immigrant population continued to grow during the entire investigation period, social resistance may have co-arisen to some degree, cancelling out positive influences of increasing immigrant populations on individual immigrants. These are only speculations of ours and await future studies to substantiate them.

## Limitations

Our sample presented much larger variation in mental health problems at the individual-level than at the municipality-level. This may have contributed to our failure to detect any effect of municipality-level proportions of immigrants on mental health problems.

This study obtained the sample from only one region in Sweden which may limit its generalizability to the entire nation. Moreover, this region has a lower rate of immigrants than some of the other more populated regions in Sweden. Also, there were huge increases in immigrants in Sweden after the investigation period. In 2015, 163,000 people applied for asylum, 35,000 of which were unaccompanied minors. Another limitation regarding the generalizability of our study findings pertains to our use of a single mental health indicator (i.e., psychosomatic symptoms). The findings of the current study cannot be generalizable to other types of mental health issues.

Due to our measurements of adolescents’ immigrant status, we were incapable of capturing some heterogeneities within immigrant population such as their countries of origin and reasons for migration. Indeed, in two of the municipalities located at the border to Norway, the proportion of Nordic immigrants was relatively higher than in the other municipalities some years. In addition, immigration generation may signify a distinctive set of risk factors for mental health. For example, while second-generation immigrant adolescents may be free from some traumatic experiences that first-generation immigrants may have had before or during migration, they may experience greater conflict in their acculturation process. We were unable to control for potential confounding effects that may have stemmed from these heterogeneities within the immigration population.

Economic situation was measured by adolescents’ report of unaffordability of daily leisure activities, although this did not seem to bias our results given that economic stress explained mental health problems among Swedish adolescents more consistently than economic status or parental unemployment did [18].

## Conclusion

In Sweden, immigrant status was a risk factor for mental health problems among adolescents. This indicates a need for more efforts to support immigrant adolescents. In addition, we call for continuous monitoring of immigrant adolescents’ mental health in order to identify underlying reasons for their higher levels of mental health problems in comparison to their native peers.
